# Ultrasonographic Evaluation of the Hypospadiac Penis in Children

**DOI:** 10.3389/fped.2022.932201

**Published:** 2022-07-06

**Authors:** Tariq O. Abbas

**Affiliations:** ^1^Pediatric Urology Section, Sidra Medicine, Doha, Qatar; ^2^College of Medicine, Qatar University, Doha, Qatar; ^3^Weill Cornell Medicine-Qatar, Doha, Qatar; ^4^Regenerative Medicine Research Group, Department of Health Science and Technology, Aalborg University, Aalborg, Denmark

**Keywords:** hypospadias, ultrasound, evaluation, urethral hypoplasia, penile anatomical disorders

## Abstract

**Introduction:**

Identifying key anatomical features of the hypospadiac penis is crucial to better understanding this pathology and guiding surgical reconstruction plans, thereby achieving superior functional and cosmetic outcomes.

**Objective:**

To Assess the feasibility and precision of penile ultrasonography (PUG) in determining key structural features for hypospadias cases (including distal extent of the spongiosal component of the urethral plate, to elucidate the healing process following tubularised incised-plate urethroplasty).

**Patients and Methods:**

Twenty-five children with hypospadias were assessed using PUG prior to surgical repair and then again under general anesthesia. Preoperative images were acquired using ultrasonography in sagittal and transverse planes, then later compared with anatomical findings obtained during surgical repair of urethral hypoplasia.

**Results:**

Median patient age was 1.2 years (range 0.5–12) and hypospadias types included coronal 17/25 (68%), mid-penile 5/25 (20%), and proximal penile 3/25 (12%). Distinct layers of the corpus spongiosa and mucosal layer, Buck fascia, tunica albuginea, glans, corpora cavernosa, and penile skin were delineated so that their spatial inter-relationship could be assessed. Distal extent of the spongiosal component of the urethral plate was determined by the mid-glans B-B line. The extent of urethral hypoplasia identified by PUG was relatively similar to measurements obtained intraoperatively.

**Conclusion:**

PUG is a feasible and accurate approach to evaluating penile configuration in children with hypospadias. Distal extent of the spongiosal component of the urethral plate was accurately determined, hence PUG could potentially be used to improve surgical planning and appraisal of current repair procedures.

## Introduction

Hypospadias is a common malformation of the penis in which abortive development of the ventral axis and corpus spongiosum leads to a range of short- and long-term effects (1–3). Delineating the anatomy of hypospadias is critical to better understanding this pathology. New techniques that can accurately detect and define structural features of hypospadias will help surgeons to select optimal repair methods, and thereby achieve superior functional and cosmetic outcomes for affected patients (4).

Several tools have been used to compare the anatomy of hypospadias and normal phallus, including histological staining (5), elastography (6), and magnetic resonance imaging (MRI) (7), which revealed variable nerve patterning, vascularity, fascial layers, and urethral plate features. Ultrasonography is a valuable diagnostic imaging tool because of its non-ionizing property and multiplanar capabilities, although radiation burden to the gonads is a consideration (particularly as many affected patients are children or young adults). Ultrasonography has been used to investigate urethral strictures in adults since the 1980s (8, 9). A recent study by Omran et al. (10) using penile ultrasonography (PUG) revealed that the thickness of the urethral plate and the underlying spongiosum is a determining factor for tubularised incised-plate (TIP) success.

The aim of this study was to determine the exact configuration of the spongiosa in cases of hypospadias. Distal extent of the spongiosa beneath the urethral plate was also assessed to better understand the healing process following TIP urethroplasty.

## Materials and Methods

**FIGURE 1:**
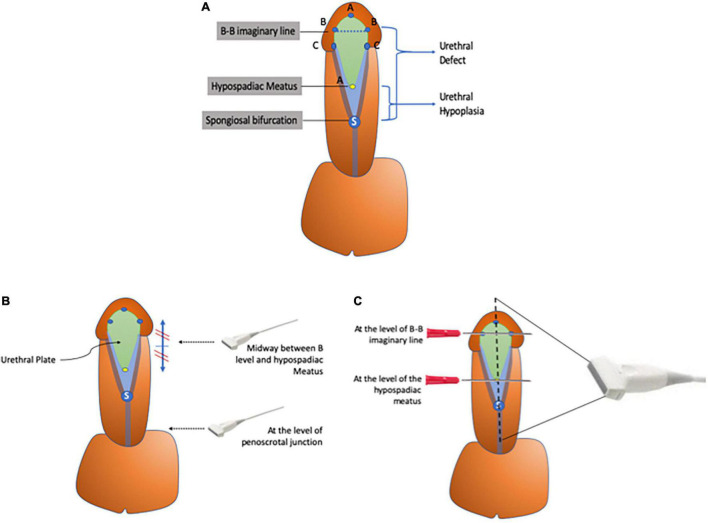
**(A–C)** Diagram representing key anatomical landmarks. **(A)** Illustration showing the different anatomical landmarks used to define urethral hypoplasia. **(B)** Two transverse images are captured: one at the peno-scrotal junction and the other midway between the hypospadias meatus and B-B line. **(C)** Longitudinal sagittal imaging over the midline of the penile shaft with two metal landmarks at the B-B line and hypospadiac meatus.

Consecutive hypospadias patients less than 18 years old were included in the study and evaluated by PUG by the surgeon to determine spatial relationships between the corpora cavernosa (CC), corpora spongiosa (CS), hypospadias meatus, and glans penis (GP). These measurements were followed by intraoperative reporting of the extent of urethral hypoplasia (distance from spongiosal bifurcation to the hypospadias meatus), and urethral defect [distance from spongiosal bifurcation to the level of the imaginary line between the two glanular knobs (B-B line)], using metal surgical calipers with no magnification, which were then compared with PUG findings (Figure 1). Ethical approval from the institutional IRB (#1837587).

Degree of penile curvature was measured under artificial erection using a mobile application (Angle Meter 360 by ALEXEY KOZLOV) using a standardized approach with the following definitions; point Y at the mid-axis of the penile shaft at the level of maximum curvature, point X at the mid-axis at the level of the tip of the glans, and point Z at the mid-axis at the level of the pubic bone (11). Patients were excluded if they presented with scrotal or perineal hypospadias, glanular hypospadias, megameatus intact prepuce, and other genital malformations, were undergoing redo surgeries, or received preoperative hormonal therapy.

Ultrasonographic examination was performed using ultrasound linear probe (Phillips IU 24 SWE–High Frequency Linear 6–12 MHz) under general anesthesia with patients in supine position and flaccid penis in the pre-pubic area immediately prior to commencing surgical repair. PUG was performed using acoustic gel and direct skin contact along the ventral surface of the penis to allow two transverse captures at the levels of the peno-scrotal junction and midway between the hypospadiac meatus and the B-B line. This was followed by capturing a longitudinal view with two metal landmarks defining the B-B level and the hypospadiac meatus (guided by their acoustic shadows). The sagittal view of the PUG is over the midline of the ventral aspect of the penile shaft and glans with a window of 3–25 mm in range in a sagittal view. The transverse view of the PUG is done with a depth window 3–25 mm.

Objective quantification of urethral plate quality was performed using the plate objective scoring tool (POST) (12, 13). Briefly, three key anatomical landmarks were marked on the glans; (A) distal extent of the urethral plate at the mucocutaneous junction, (B) glanular hillocks where the AB line starts to diverge laterally and defines the lateral borders of the urethral plate, and (C) beginning of the coronal margin where this commences along the BC line. POST ratio was calculated as follows (Equation 1):


(1) $$P\,O\,S\,T\,r\,a\,t\,i\,o=\frac{A\,B\,d\,i\,s\,t\,a\,n\,c\,e}{B\,C\,d\,i\,s\,t\,a\,n\,c\,e}$$

Descriptive statistics were used to summarize and determine the sample characteristics and distribution of various considered parameters related to ultrasonographic evaluation of the hypospadias penis in children. The normally distributed data and results were reported with mean and standard deviation (SD), whereas median and interquartile range (IQR) were used in non-normal data distribution. Categorical data were summarized using frequencies and percentages. General and penile anthropometric parameters values of the studied patients measured across three independent groups (i.e., distal, mild, and proximal penile groups) were compared using Pearson Chi-square or Yates corrected Chi-Square tests as appropriate (for qualitative variables) and one-way analysis of variance (ANOVA) or Kruskal–Wallis test as appropriate (for quantitative outcome measures). All statistical *p*-values presented were two-tailed, and *p*-values < 0.05 was considered as statistically significant. All Statistical analyses performed using statistical packages SPSS version 27.0 (Armonk, NY: IBM Corp).

## Results

Between January 2022 and May 2022, a total of 25 children with coronal (17/25; 68%), mid-penile (5/25; 20%), or proximal hypospadias (3/25; 12%) were included in this study. General characteristics of the study cohort are summarized in Tables 1, 2. Although was not statistically significant, the penile curvature (>20°) occurred more often the more proximal the hypospadias meatal position. However, the absolute degree of the curvature and POST score strongly correlated with meatal position (*p* < 0.0001, 0.003, respectively). While determination of the extent of urethral hypoplasia was not precise by physical examination, both intraoperative and PUG assessment were relatively more similar.

**TABLE 1:** General and penile anthropometric values of study patients.

	Distal penile	Mid penile	Proximal penile	Test statistic value	*P*-value
Number	17	5	3		
Age (months)	17 (range from 8 to 39)				
POST score	1.1 ± 0.12	0.9 ± 0.22	0.7 ± 0.38	ANOVA *F* = 7.51	0.003
Curvatures (>20)	5/17	4/5	3/3	χ^2^ = 4.14	0.126
Curvature degrees (*when available*)	30 ± 10	40 ± 5	60 ± 15	ANOVA *F* = 12.36	<0.0001
Starched penile length (mm)	57 ± 12	52 ± 9	49 ± 10	ANOVA *F* = 0.86	0.437

**TABLE 2:** The extent of the urethral hypoplasia proximal to the hypospadias meatal opening determined using either physical examination, PUG, and intraoperatively.

	Distal penile	Mid penile	Proximal penile	Test statistic value	*P*-value
By physical exam	3 ± 3	4 ± 4	5 ± 2	ANOVA *F* = 0.62	0.547
Using PUG	3 ± 3	6 ± 4	10 ± 2	ANOVA *F* = 7.14	0.004
Intraoperatively	4 ± 3	7 ± 5	9 ± 2	ANOVA *F* = 3.67	0.042

**FIGURE 2:**
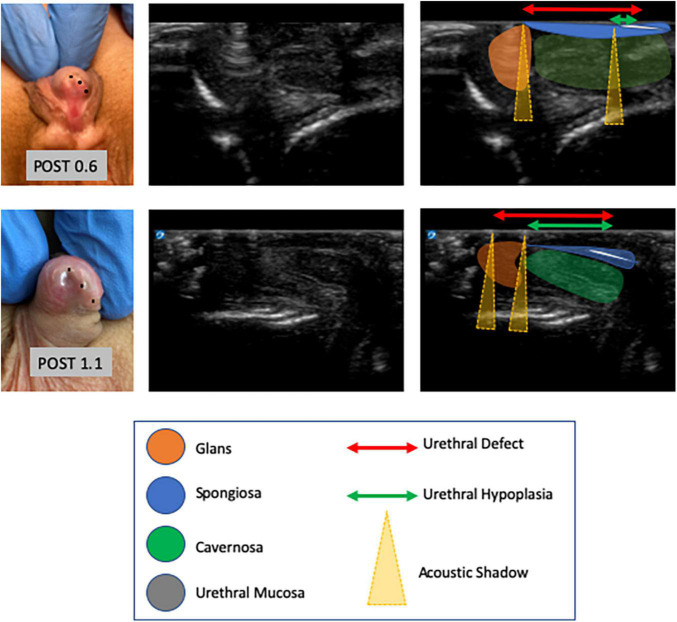
Images of hypospadias cases with their corresponding POST values (left column) and subsequent PUG images (middle column) with annotations shown in the third column (right). To better define the extent of urethral under-development, we defined urethral hypoplasia as the distance from the spongiosal bifurcation to the hypospadias meatus, whereas urethral defect was defined as the distance from the spongiosal bifurcation to the B-B line. We observed that a longer urethral defect was detected in more severe cases of hypospadias, when stratified according to meatal position, and that longer urethral defect correlated with lower POST values (*p* = 0.001).

The CC is a relatively hypoechoic cylindrical structure (14) lined by the thin hyperechogenic membrane of the tunica albuginea. The ventromedial structure of the corpus spongiosum surrounds the urethra and is more echoic than the CC, but is similarly covered by the tunica albuginea. Buck’s fascia is superficial to the tunica albuginea and overlies all of the structures described above (Figure 2).

## Discussion

In this study we show that PUG is a feasible and useful tool that enables high precision evaluation of hypospadias in children. PUG successfully determined the different structures within the hypospadias penis as well as the two-dimensional spatial relationships between them. This methodological study of feasibility and sensitivity of ultrasonography of the penis in cases of hypospadias in children has several clinical implications. It can facilitate the preoperative risk stratification based on defining the exact of the urethral hypoplasia (15) and thus predicting the potential for associated penile curvature and potential surgical technique. Furthermore, it can help in the decision-making processes of preoperative parental counseling.

Tubularised incised-plate urethroplasty was popularized by Snodgrass in 1994, since this technique made tubularisation of shallow and narrow plates more amenable (16), although complication rates can still exceed 20% (17). The dorsal plate incision creates a raw area that heals by secondary intension, necessitating deposition of a thin layer of granulation tissue and epithelial creeping to cover this, thereby conferring susceptibility to fibrosis and stiffness of the neourethra (17–21) (often overcome by performing several sessions of post-operative urethral dilatation).

Urethral wound healing is perceived to be a multifaceted and active process that includes multiple different cell types at each stage (22). The initial stage of wound healing is hemostasis, followed by the inflammation stage, the proliferative stage, and finally the maturation stage. The inflammatory state that follows hemostasis is an essential and vital aspect of wound healing, during which immune cells migrate into the wound field to eliminate microorganisms and clear tissue debris. This immune infiltrate produces a range of cytokines and chemokines that additionally stimulate fibroblasts and epithelial cells to further promote wound healing (23). When the shielding barrier of uroplakin is dysfunctional, as occurs in the early phases of urothelial regeneration, cytotoxic agents can bind to the anionic surface of underlying tissues (24). Moreover, it was recently shown that interstitial fluid flow plays a significant role in the wound healing response, suggesting that any leakage of urine from within the urinary tract may provoke local fibrosis and delay healing. Therefore, the healing of the urethral wound which happens during the urethroplasty depends basically on the spongiosal component of the urethral wall. Thus, exploration of the presence, thickness, robustness of urethral spongiosa in cases of hypospadias could potentially facilitate the understanding the healing process of the urethroplasty following hypospadias repair. We observed that distal extent of the spongiosal component of the urethral wall is situated at the level of the mid-glans B-B line, consistent with a previous report by Ozbey et al. (25). The urethral plate when incised can therefore distinct healing processes within the area distal to the end of the underneath spongiosa. Healing of the neourethra after TIP urethroplasty could potentially be improved *via* formation of a neourethra that is wholly lined with epithelium. To achieve this, grafting of the dorsal incised (GTIP) area using the inner prepuce has been described by several surgeons (26–28). However, healing of the grafted TIP process is time-consuming and predisposed to complications, such as graft detachment due to catheter friction, infection, and also contracture.

Initial experiences with ultrasound evaluation of the urethra in the late 1980s were described separately by McAninch et al. (8) and Merkle and Wagner (9). Since then, ultrasonography has shown the ability to reveal urethral strictures and abnormalities within the periurethral territories (29). Current linear transducers benefit from high frequency, which increases the resolution on ultrasound images. The penis is predominantly a superficial organ, and for the most part easily examined by palpation or with ultrasound. Recent advances in ultrasonographic techniques (such as extended-field-of-view imaging) further add to utility of this technique, and are particularly useful for demonstrating pathology to non-radiologists (30). Preoperative ultrasonography performed in the clinic setting for the diagnosis and characterization of anterior urethral strictures was safe and feasible, offering a useful alternative to retrograde urethrogram (31). Using this technique, it is possible to demonstrate the anatomy of the hypospadiac penis in transverse longitudinal scanning planes, which could prove invaluable for surgical planning. Indeed, a recent study by Buckley et al. (32) demonstrated that ultrasonography of the urethra can directly influence the reconstructive operative approach in 45% of cases undergoing anterior urethroplasty. The transverse section images are particularly useful to assess thickness of the spongiosa component underneath the urethral plate, while the longitudinal aspect provides a panoramic view of the penile urethra (which is particularly helpful when diagnosing urethral structure after hypospadias repair). We did not use any lumen-filling agent and relied on the hyper-echogenic urethral mucosa to define extent of the lumen, thereby avoiding technical difficulties and possible artifacts associated with these agents. This approach has the advantage of being noninvasive, widely available, and avoids exposure to ionizing radiation (which is of particular importance in children). These important anatomical findings could help reconstructive surgeons to understand complications resulting from hypospadias repair and assist their decision making processes (33).

The relatively small number of patients examined in this study could be considered a limitation, as was the involvement of a single measuring surgeon, which prevents us from assessing inter-observer variability. Although sequential performance of eye, ultrasonographic and intraoperative assessment could potentially be considered adequate for such feasibility study, no formal blinding scheme was conducted. Future studies that consider larger numbers of patients and evaluating surgeons interobserver variability and reproducibility will therefore be required to enforce these findings.

## Conclusion

We showed that PUG is a feasible and accurate technique to evaluating penile configuration in children with hypospadias. Distal extent of the spongiosal component of the urethral plate was precisely defined, hence PUG could potentially be used to improve surgical planning and appraisal of current repair procedures.

## Data Availability Statement

The raw data supporting the conclusions of this article will be made available by the authors, without undue reservation.

## Ethics Statement

The studies involving human participants were reviewed and approved by the Institutional Review Board, Project # 1837587, Sidra Medicine, Doha, Qatar. Written informed consent to participate in this study was provided by the participants’ legal guardian/next of kin.

## Author Contributions

TA conceptualized the idea, collected the data, and analyzed and wrote the manuscript.

## Conflict of Interest

The author declares that the research was conducted in the absence of any commercial or financial relationships that could be construed as a potential conflict of interest.

## Publisher’s Note

All claims expressed in this article are solely those of the authors and do not necessarily represent those of their affiliated organizations, or those of the publisher, the editors and the reviewers. Any product that may be evaluated in this article, or claim that may be made by its manufacturer, is not guaranteed or endorsed by the publisher.
